# Virulence Factors and Phylogeny of *Staphylococcus aureus* Associated With Bovine Mastitis in Russia Based on Genome Sequences

**DOI:** 10.3389/fvets.2020.00135

**Published:** 2020-03-25

**Authors:** Ksenia Fursova, Anatoly Sorokin, Sergey Sokolov, Timur Dzhelyadin, Irina Shulcheva, Margarita Shchannikova, Daria Nikanova, Olga Artem'eva, Natalia Zinovieva, Fedor Brovko

**Affiliations:** ^1^Laboratory of Immunochemistry, Shemyakin and Ovchinnikov Institute of Bioorganic Chemistry of the Russian Academy of Sciences, Pushchino, Russia; ^2^Laboratory of Cell Genome Functioning Mechanisms, Institute of Cell Biophysics of the Russian Academy of Sciences, Pushchino, Russia; ^3^Laboratory of Plasmid Biology, G.K. Skryabin Institute of Biochemistry & Physiology of Microorganisms of the Russian Academy of Sciences, Pushchino, Russia; ^4^Laboratory of Microbiology, L.K. Ernst Federal Science Center for Animal Husbandry, Moscow, Russia

**Keywords:** *S. aureus*, virulence genes, enterotoxins, cytotoxins, multidrug resistance, biofilms, mastitis

## Abstract

*Staphylococcus aureus* is a causative agent of different infectious processes, food poisoning, and autoimmune disorders. The horizontal transfer of pathogenic strains can occur from animal to human under both house and farm conditions, and the spread of strains with antibiotic resistance is an existing problem. In addition to the spread of antibiotic-resistant strains in clinics, this problem also exists in veterinary medicine. It is especially important to monitor antibiotic resistance on farms where antibiotics are the standard treatment of animals, which may trigger the spread of antibiotic-resistant strains among animals and to the human population, and these strains can also be distributed in milk products produced by these farms (milk, cheese, and butter). In this work, we investigated 21 *S. aureus* isolates using whole-genome sequence analysis and tried to establish a relationship between these isolates with the development of bovine mastitis in seven regions of Western Russia. An *S. aureus* virulence profile was identified. We identified two groups of *S. aureus* associated with subclinical mastitis, namely, the enterotoxin-positive and enterotoxin-negative groups. The most prevalent factor associated with bovine mastitis in Russia was cytotoxins, including hemolysins and leukocidins. Multidrug resistance strains were investigated, and antibiotic resistance genes were identified. We identified *S. aureus* ST 97 type as the most common type in the regions in Western Russia. To the best of our knowledge, this is the first in-depth study of a range *S. aureus* isolates originating from cattle infections in Russia.

## Introduction

Bovine mastitis is a widespread infectious disease that lacks a fully investigated etiology. It is considered that various factors, such as the environment, defects in milking techniques, lactation stage, or different infections, can trigger mastitis development (1). Infectious mastitis has a viral or bacterial origin. The most common bacterial agents are *Staphylococcus, Streptococcus*, coliforms, *Micrococcus*, and *Bacillus* species. These are opportunistic microorganisms that can switch to a pathogenic form and use various virulence factors to cause various pathologies in both animals and humans. Antibiotic therapy is actively carried out to prevent infection, which in turn triggers the problem of antibiotic resistance in microorganisms. At present, the methicillin-resistant *S. aureus* (MRSA) strains, including those of both animal and human origins, are prevalent (2). According to recent data, the frequency of MRSA cases has decreased, whereas the cases associated with methicillin-susceptible *Staphylococcus aureus* (MSSA) have been detected with increasing frequency (3).

In recent years, the frequency of the confirmed transition of antibiotic-resistant strains from animal to human populations is rapidly growing. Therefore, the study of the microflora of animals in close contact with humans is relevant (4–7).

This work aimed to investigate the virulence profile of *S. aureus* isolates obtained from cow's milk in Russia and to determine their potential involvement in the induction of mastitis in cows.

In this report, the whole-genome sequencing (WGS) characteristics of 21 *S. aureus* isolates from raw cow's milk samples from different regions of Russia are presented. Furthermore, we investigated the virulence factors and antibiotic resistance properties of the isolates. All the isolates were identified as methicillin-susceptible *S. aureus*, and multiple drug resistance was determined for several samples.

This study confirms the need for a systematic approach in modern studies of animal microflora because it can spread to the human population.

## Materials and Methods

### Sample Collection

#### *Staphylococcus aureus* Isolation

Raw milk samples were collected from cow farms from different regions of Russia in 2018. The milk samples were taken from each cow during aseptic milking and placed in sterile containers. The samples were transported to the laboratory at a temperature of +4°C within 2 h or frozen at −20°C. Salt meat broth (HiMedia Laboratories Pvt., Ltd, Mumbai, India) was inoculated with the milk samples at a ratio of 1:9 and stored at 37°C for 18–24 h. Internationally recognized traditional phenotypic methods, such as Gram-stained colony microscopy, growth in Baird–Parker agar (HiMedia Laboratories Pvt., Ltd.), hemolysis on azide blood agar Pronadisa (Condalab, Madrid, Spain), plasma coagulation, and biochemical identification, were applied to all the isolates. The strains were stored in trypticase soy broth (TSB) (Merck, Darmstadt, Germany) with 30% sterile glycerine at −18°C.

#### DNA Extraction

*Staphylococcus aureus* isolates were cultivated in TSB at 37°C on an orbital shaker for 14–16 h. The culture liquid was centrifuged at 4,000 g for 5 min at 4°C, and the cell pellet was used for DNA extraction.

Twenty-one isolates were selected for WGS. DNA samples were extracted using the Lysostaphin and GenElute Bacterial Genomic DNA kit (Sigma–Aldrich, Merck). DNA was analyzed by electrophoresis in a 1% agarose gel stained with ethidium bromide (5 μg/mL).

### Genome Sequencing and Data Preprocessing

#### Genome Sequencing

Sequencing was performed using an Illumina inc., San Diego, USA with MiSeq Reagent Kit v3 chemicals. The quality of the sequencing results was assessed by FastQC, and the sequences were trimmed with Bjorn Usadel Lab, Aachen, Germany to keep quality above 25.

#### Genome Annotation and Deposition

The trimmed reads were uploaded to the PATRIC server (8), and the genomes were assembled and annotated with the PATRIC Comprehensive Genome Analysis pipeline (9). Only the genomes placed close to *S. aureus* by PATRIC Phylogenetic Analysis tree were retained for further analysis. All the genomes were deposited to GenBank.

### Virulence Gene Identification

The presence and allele of the antimicrobial resistance and virulence genes were evaluated by SRST2 (10). Two resources, namely, the ARG database (11) and ResFinder (12), were used for antimicrobial resistance, and VFDB (13) was used to identify virulence and pathogenicity gene annotation. The antimicrobial resistance database files were downloaded together with SRST2 software, and the *S. aureus* subset of VFDB was prepared as described in the SRST2 manual. The default parameter values were used for analysis in accordance with suggestions in the SRST2 manual.

### Antibiotic Resistance Investigation

Antibiotic susceptibility testing was performed using the disc diffusion method for the following antibiotics (HiMedia Laboratories Pvt., Ltd.) in Mueller-Hinton Agar (HiMedia Laboratories Pvt., Ltd.): penicillin (10 IU), oxacillin (1 μg), gentamicin (10 μg), erythromycin (15 μg), and ciprofloxacin (5 μg). Methicillin-resistant *S. aureus* isolate screening was performed using oxacillin (1 μg/disc). The dishes with inoculated cultures were incubated under aerobic conditions at 37 ± 1°C for 18–24 h. The sensitivity was evaluated by the diameters of the zones of inhibition in accordance with the Performance Standards for Antimicrobial Disc Susceptibility tests (14); EUCAST, http://www.eucast.org.

### Phylogenetic Characterizations

#### SNP Phylogeny of Whole-Genome Sequences

The Single nucleotide polymorphism (SNP) tree was constructed using CSI Phylogeny 1.4 (Center for Genomic Epidemiology) using default settings and excluding heterozygous SNPs. To identify SNPs, all the input sequences were mapped to the *S. aureus* subspecies *aureus* LGA251 genome as the reference (NCBI accession no. FR821779.1) and screened for the relevant nucleotide variations as previously described (15). The following criteria for high-quality SNP calling and filtering were chosen: (i) a minimum depth of 10× at SNP positions; (ii) a minimum relative depth of 10% at SNP positions; (iii) a minimum distance of 10 bp between SNPs; (iv) a minimum SNP quality of 30; (v) a minimum read mapping quality of 25; and (vi) a minimum *Z* score of 1.96. Site validation for each SNP position was performed. SNPs that failed the necessary requirements were excluded in the final analysis. Based on the concatenated alignments of high-quality SNPs, maximum likelihood trees were created using MEGA7 (16).

#### Multilocus Sequence Typing

SRST2 (10) software was used to map the trimmed reads to the multilocus sequence typing (MLST) database (17). Strain definition files for *S. aureus* were downloaded as described in the SRST2 manual, and the analysis was performed with SRST2 default parameter values.

#### Genome-to-Genome Comparison

The genomes of the *S. aureus* isolates, which had similar biochemical and genetic characteristics, were compared for a genome distance (18) using programs available online (http://ggdc.dsmz.de/). DNA-DNA hybridization values were measured based on formula 2.

### Ethics Statement

All the animal manipulations were performed in accordance with the Instruction for Diagnostics, Therapy and Prevention of Mastitis (no. 13-5-2/1948; http://gov.cap.ru/home/65/aris/bd/vetzac/document/371.html) and approved by the Department of Veterinary, Ministry of Agriculture of the Russian Federation. In addition, all the procedures followed the Guidelines for the Microbiological Study of Milk and the Secretion of the Udder of Cows for the Diagnosis of Mastitis (https://standartgost.ru/g/pkey-14293740821), Ministry of Agriculture of the Russian Federation.

## Results

### *S. aureus* Isolation

**Figure 1:**
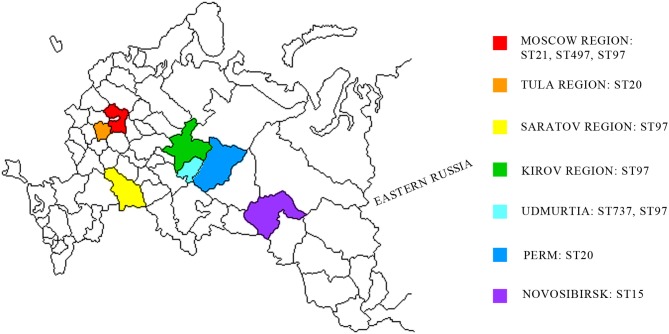
Geographic distribution of *S. aureus* strains associated with subclinical mastitis in the western region of Russia. Studied regions are shown in different colors. MLST distribution of *S. aureus* isolates are shown in studied regions.

Raw milk samples were collected from farms in different regions of Russia (Figure 1). Pure *S. aureus* cultures were obtained from raw cow's milk. The purified DNA samples of 21 isolates (data not shown) were used for WGS.

The WGS data were analyzed by the following two separate pipelines: (i) SRST2 software was used to map the reads to special gene databases, such as MLST for strain annotation, ARG for antibiotic resistance genes, and VFDB for virulence factors; and (ii) the PATRIC Comprehensive Genome Analysis pipeline was used to assemble and annotate the genomes. The overall results of both approaches agreed with each other. For the analysis of virulence factors, however, we used SRST2 data, as these data provide depth and allele information.

### Accession Number(s)

All the genomes were deposited to GenBank with the following GenBank accession numbers: WIPL00000000, WIPM00000000, WIPN00000000, WIPO00000000, WIPP00000000, WIPQ00000000, WIPR00000000, WIPS00000000, WIPT00000000, WIPU00000000, WIPV00000000, WIPW00000000, WIPX00000000, WIPY00000000, WIPZ00000000, WIQA00000000, WIQB00000000, WIQC00000000, WIQD00000000, WOUL00000000, and WNKR00000000.

### Analysis of the Virulence Genes

**Figure 2:**
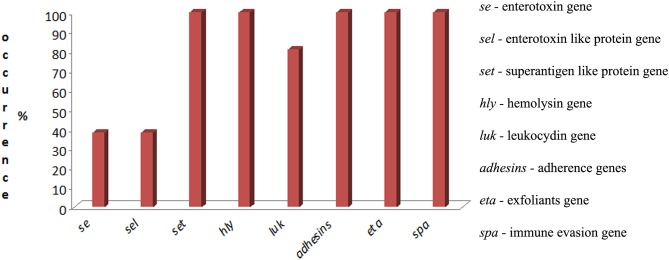
Occurrence of virulence genes of *S. aureus* isolated from raw cow's milk with subclinical mastitis in the western region of Russia.

Twenty-one *S. aureus* isolates were analyzed. We identified genes from the following seven virulence families: enterotoxins, enterotoxin-like proteins, superantigen-like proteins, cytotoxins (including hemolysins and leukocidins), adherence, immune evasion, and exfoliatins (Supplementary Table 1). The occurrence of virulence genes is presented in Figure 2.

None of the investigated strains contained genes encoding the classic enterotoxins from *sea to see*. Moreover, 38% of the isolates (*n* = 8) were classified as toxicogenic by enterotoxins *seg* and *sei*. Genes encoding the staphylococcal enterotoxin-like proteins (*sel*), namely, *selm, seln, selo*, and *selv*, were identified in this group, which consisted of 8 isolates (38%).

Genes encoding superantigen-like proteins (*set*) were detected in all the isolates (*n* = 21). All the isolates (*n* = 21) harbored genes encoding hemolysins (*hla, hlb, hlg*, and *hld*). Leukocidin genes, including *lukE/D* and *lukM/F*, were detected in 80.9% (*n* = 17) and −4.7% (*n* = 1) of isolates, respectively, but the *luk S/F* genes were not identified.

Genes encoding adhesins, namely, *icaA, icaB, icaC*, and *icaD*, were identified in 100% of the isolates (*n* = 21); and *fnbA* and *fnbB* were identified in 42.8% (*n* = 9) of the isolates. Spa genes were characteristic for all the *S. aureus* isolates (*n* = 21). Surprisingly, all the isolates (*n* = 21) had *eta* genes, but *etb* and *etc* were not identified.

### Antibiotic Resistance of the *S. aureus* Isolates

Eight isolates that harbored staphylococcal enterotoxin genes (*seg* and *sei*) were selected for the study of antibiotic resistance. The antibiotic resistance results are shown in Table 1. The investigated isolates were resistant to erythromycin (100%), gentamicin (75%), penicillin (62.5%), and ciprofloxacin (25.5%). Among all the strains, the highest susceptibility was observed with respect to oxacillin (100%). Strains 615 and 1703 were resistant to gentamicin and erythromycin. Strains 88, 1838, and 1839 showed simultaneous penicillin, gentamicin, and erythromycin resistance. Strains 74 and 8656 had multiple resistance.

**Table 1:** Antibiotic resistance of enterotoxigenic *S. aureus* isolates.

**Isolate**	**Penicillin, 10 IU**	**Oxacillin, 1 μg**	**Ciprofloxacin, 5 μg**	**Gentamicin, 10 μg**	**Erythromycin, 15 μg**	**Detected antibiotic resistance genes**
70	S	S	S	S	R	*blaZ, ermC, aac(3), aph(2), norA*
74	R	S	R	R	R	*blaZ, ermC, aac(3), aph(2), norA*
88	R	S	S	R	R	*blaZ, ermC, aac(3), aph(2), norA*
1838	R	S	S	R	R	*blaZ, aac(3), aph(2)*
1839	R	S	S	R	R	*blaZ, aac(3), aph(2)*
615	S	S	S	R	R	*blaZ, aac(3), aph(2), norA*
1703	S	S	S	R	R	*blaZ, aac(3), aph(2), norA*
8656	R	S	R	S	R	*aac(3), aph (2), norA*

*R, resistant; S, susceptible; NI, none identified*.

The following genes encoding antibiotic resistance proteins were identified: *blaZ, ermC, aac* *(**3**), aph* *(**2**)*, and *norA* (Table 1).

### Phylogeny

#### SNP Phylogeny

**Figure 3:**
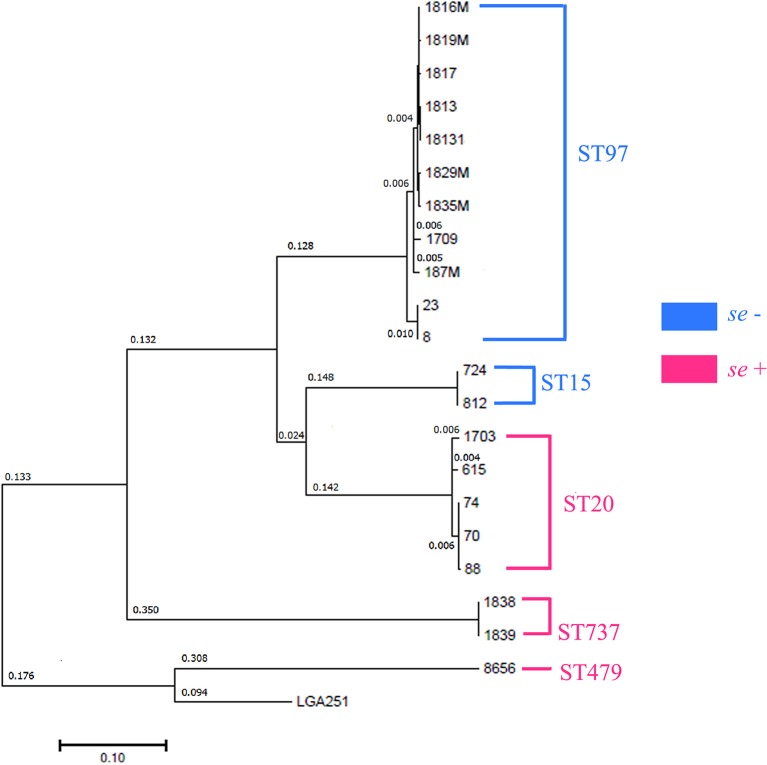
Phylogenetic analysis of *S. aureus* isolates by the maximum likelihood method. The log likelihood of the tree is (−233,811.0045). The tree is drawn to scale with branch lengths measured in the number of substitutions per site. All the positions containing gaps and missing data were eliminated. There were 39,835 positions in the final dataset. Evolutionary analyses were conducted in MEGA7. Distances >0.001 are shown. Sequence types (MLST) are shown in clusters.

SNP analysis (https://cge.cbs.dtu.dk/services/CSIPhylogeny/) was conducted to study the differences in the genomes among the isolates and to determine their potential relationships (Figure 3). The studied isolates formed the following five clusters: (i) individual strain 8656; (ii) strains 1838 and 1839; (iii) strains 70, 74, 88, 615, and 1703; (iv) strains 812 and 724; and (v) strains 1817, 1816, 1813, 1829M, 1819M, 1835M, 18131, 187M, 1709, 23, and 8. Isolate 8656 was the most closely related to the reference genome LGA251 identified from bovine population (GenBank accession no. FR821779.1).

Isolates 70, 74, and 88 differed with 0.01-nucleotide substitution per site, but they shared different phenotypes.

#### MLST

To determine the sequence type (ST) of the isolated strains, MLST analysis was performed *in silico* using the MLST database (https://cge.cbs.dtu.dk/services/MLST/).

Using MLST, we detected the following five STs: ST20, ST479, ST737, ST97, and ST15. ST97 was the most common type that was detected in the Moscow, Kirov, Udmurtia, and Saratov regions (Figure 1, Supplementary Table 1). The isolates were categorized in the following STs: isolates 70, 74, 88, 615, and 1703 belong to ST20; isolates 724 and 812 belong to ST15; isolates 1838 and 1839 belong to ST737; and isolate 8656 belongs to ST479.

Five SNP clusters were found to correlate both with genotypic (MLST) and with phenotypic (se) traits (Figure 3).

#### Genome-to-Genome Comparison

Genome-to-genome comparison was provided for the isolates with high identity according to their biochemical and genetic properties or pairs from one region.

The calculation of the intergenomic distance (Table 2) showed that genome pairs 1838–1839 (distance: 0.0001), 812–724 (distance: 0.0002), 70–88 (distance: 0.0000), and 23–8 (distance: 0.0000) belong to the same genomic subspecies. Pairs 70–74 (distance: 0.0014), 74–88 (distance: 0.0014), and 615–1703 (distance: 0.0008) were slightly spaced from each other.

**Table 2:** Genome-to-genome comparison; pairwise analysis of *S. aureus* genomes.

**Genome 1**	**Genome 2**	**DDH, %**	**Model CI, %**	**Distance**	**Prob. DDH >79%**	**G+C difference**
1838 (WIPQ00000000)	1839 (WIPR00000000)	100.00	[100–100]	0.0001	80.27	0.00
724 (WIPV00000000)	812 (WIPU00000000)	99.90	[99.9–100]	0.0002	80.15	0.01
70 (WIPM00000000)	88 (WIPN00000000)	100.00	[100–100]	0.0000	80.31	0.01
23 (WIPS00000000)	8 (WIPT00000000)	100.00	[100–100]	0.0000	80.32	0.03
70 (WIPL00000000)	74 (WIPM00000000)	99.2	[98.8–99.5]	0.0014	78.87	0.01
74 (WIPM00000000)	88 (WIPN00000000)	99.3	[98.9–99.5]	0.0013	78.95	0.03
615 (WIPP00000000)	1703 (WOUL00000000)	99.6	[99.3–99.8]	0.0008	79.47	0.05

*DDH, DNA-DNA hybridization; CI, confidence interval*.

## Discussion

Most often, bovine mastitis is associated with superantigens or enterotoxins produced by *S. aureus* (19–21) or coagulase-negative staphylococci (22). Staphylococcal enterotoxin C is the most common cause of bovine mastitis (23–25). According to other authors, one of the main enterotoxin genes of *S. aureus* isolated from cows with mastitis is *sea*. This gene occurs at rates ranging from 23.6 to 50% (21). Franck et al. (26) showed that mastitis in human females is also associated with the *sec* and *sell* genes, which are located in pathogenicity islands and are potentially transferred from cows to humans. For this reason, we conducted molecular characteristic analyses of the virulence potential of staphylococcal isolates from cow's milk to study mastitis in cows associated with *S. aureus* in Russia.

Twenty-one *S. aureus* samples were isolated from raw cow's milk with subclinical mastitis. By analyzing previous studies on *S. aureus* associated with intramammary infections in dairy cows of different geographical regions (4, 27–29) and using the virulence factor database (VFDB; http://www.mgc.ac.cn/cgi-bin/VFs/genus.cgi?Genus=Staphylococcus), we selected seven types of virulence genes because of their known pathogenic properties that may be associated with mastitis in Russia. Eight *S. aureus* isolates harbored the staphylococcal enterotoxin genes, *seg* and *sei*. Other genes encoding the classic *se* from *sea* to *see* were not identified. In previous studies, the occurrence of the *seg* and *sei* genes varied. Mello et al. (22) detected the *seg* gene only for 1% of *S. aureus* and 2% for central nervous system isolates, and *sei* was detected for 11 and 3% for *S. aureus* and coagulase-negative isolates, respectively. In contrast, Srinivasan et al. (30) detected *seg* and *sei* with higher frequencies (60.3% for *sei* and 24.4% for *seg*).

These *seg/sei*-positive isolates harbored genes encoding enterotoxin-like proteins, namely, *selm, seln, selo, selu*, and *selv*, which was not surprising because of their association with the enterotoxin gene cluster (egc) that is organized as an operon (31). The cluster consists of two enterotoxin genes (*seg* and *sei*), three enterotoxin-like genes with proven superantigenic activity but not emetic properties (*selo, selm*, and *seln*), and two pseudogenes (ϕ*ent1* and *2*). In turn, SEU is the fusion product of ϕent1 and ϕent2, which results from a 15-nucleotide deletion in ϕent1 (32).

The family of superantigen-like proteins (*set*) was identified as a common gene group in the analyzed isolates. These genes are located in the *S. aureus* genome by pathogenicity islands (33) and encode secreted virulence factors related to the host–pathogen interaction process (34).

The next antigenic target of our analysis was a group of cytotoxins, including hemolysins and leukocidins. We observed high occurrence of these genes. Cytotoxin genes were identified at high levels. Namely, genes of hemolysins (*hla, hlb, hld, hlg*) were detected in all investigated regions of Russia. Genes of leukocydins *lukE/D* in 80.9% of isolates and *lukM/F* in only one isolate (4.7%), although *lukM/F* is associated with zoonotic infections, and the LukM/F produced during the course of infection and the LukM/F levels in milk are associated with the severity of mastitis (27). In our previous work, we detected the following cytotoxin genes with a high frequency: *lucS* (46.6%), *hla* (70%), and *hld* (78.3%) (35). The high level of *hly* detection (up to 93%) has been observed by others (29). These agents are known for their role in the development of mastitis. Leukocidins may cause the lysis of leucocytes in bovine mastitis (36) and provide a protective masking effect for *S. aureus* in the host (37). α-Hemolysin induces gangrenous mastitis, which damages smooth muscle, restricting blood supply to mammary tissue (38).

Interestingly, 100% of the isolates from different regions of Russia harbored the *eta* gene. However, this finding requires verification by further analysis. In recent work, Schmidt and coauthors did not identify *eta, etb*, or *etd* in bovine isolates, and they reported that only one human *S. aureus* isolate tested positive for *eta* (4). It is possible that our finding in the current study may be a result of the coincidence of mastitis with different dermatitis conditions, for example, hock lesions (39) or udder cleft dermatitis (40, 41). Udder cleft dermatitis is a pathology lacking a clear etiology, but one of the bacterial inducers can be *S. aureus*. Moreover, some cases of sheep mastitis have been associated with the exfoliative toxin D–like protein (42).

We identified genes of two protein families with high occurrence. All the isolates harbored genes encoding intercellular adhesins encoding intercellular adhesion operon (*Ica*ADBC) (43), and 42.8% of isolates harbored genes encoding fibronectin-binding proteins (44, 45). Usually, these genes are associated with biofilm formation, which is associated with antibiotic resistance. In particular, fibronectin-binding proteins A and B mediate the adhesion of *S. aureus* to fibrinogen, elastin, and fibronectin, thus providing *S. aureus* the ability to invade endothelial cells without requiring additional factors, although slowly and inefficiently, through actin rearrangements in host cells (46) (https://www.uniprot.org/uniprot/P14738). Furthermore, *S. aureus* surface protein A may participate in biofilm formation (47).

Investigation of antibiotic resistance showed that eight of the studied isolates were resistant to different classes of antibiotics. Moreover, isolates 74, 88, 1838, 1839, and 8656 were identified as Multiple drug resistance (MDR), as they were resistant to three or more antimicrobial classes according to the guidelines recommended by the joint initiative of the European Center for Disease Prevention and Control and the Centers for Disease Control and Prevention (48). Whole-genome sequencing analysis confirmed the diversity of antibiotic resistance genes.

Antibiotic treatment of bacterial infections may cause the development of MDR. This problem has become widespread both in veterinary and human medicine. The drug resistance of *S. aureus* has multiple origins, such as the use of efflux pumps, increased expression of target proteins, or mutation of target proteins (49). In the present study, neither the *mecA* nor *mecC* genes were detected in any isolate, whereas all the isolates (excepted 8656) harbored the *blaZ* gene. Multiple and methicillin resistance can be associated with biofilm formation of *S. aureus*. Previous studies have reported that biofilm formation is a common characteristic of MRSA strains (50). Isolates 70, 74, and 88 also had the *ermC* gene, which is responsible for their resistance to erythromycin. We observed the presence of *aac* *(**3**)* and *aph* *(**2**)*, which confirmed resistance to gentamicin (51). The *norA* gene was detected in the common group, and it may provide non-specific resistance to different antibiotics.

As described in many studies, the specific strains of *S. aureus* that are associated with mastitis vary across herds (28, 52, 53). Strains with identical genotypes may possess characteristics that provide some advantages for their survival in the environment and to colonize the udder (22). We performed phylogeny analysis and multilocus typing of isolates, and we detected five clusters. As shown at Figure 1, ST97 was identified to be geographically close to other regions, such as Kirov and Udmurtia, but not close to outlying regions, such as Moscow and Saratov. In our previous work, ST97 isolates were identified in farms of Central Russia (54). Based on the data presented on the MLST resource, ST97 was present in the remainder of the group. This type is quite common, as it is found among isolates of China, Spain, Japan, the Netherlands, Brazil, and other countries (https://pubmlst.org/bigsdb?db=pubmlst_saureus_isolates). In addition to being detected in the present study, ST20 is the most widely represented among the isolates of China and Japan followed by ST737 in Turkey and ST479 in the Netherlands and USA (https://pubmlst.org/bigsdb?db=pubmlst_saureus_isolates).

By summarizing these phylogeny results with virulence data, we identified the following two groups: *se*-negative (formed by ST15 and ST97) and *se*-positive (formed by ST20, ST737, and ST479). The *se-*negative group was composed of the samples isolated from regions located in different geographic and climatic regions (Kirov, Saratov, Novosibirsk, and partially Moscow). The *se-*positive group was isolated from regions that geographically pairwise border Udmurtia and Perm and Moscow and Tula and that belong to the same climatic zone. In addition, an animal transfer between farms of these neighboring regions is possible. These factors may account for the spreading of enterotoxigenic strains in these regions.

According to Grunert, infection in a herd can be linked to specific strain genotype and often results in one or a few dominant clones within a herd, indicating transmissibility between animals and the preference of particular pathotypic traits (55). We detected high genetic similarity within groups and the similarity of these groups to ST types, which allowed us to hypothesize that isolates of the phylogenetic group with one ST type may belong to the one genomic subspecies. In addition, such isolate pairs, namely, 1838–1839 (Udmurtia) and 615 (Perm)−1703 (Moscow), had similar antibiotic resistance in their pairs. A genome-to-genome comparison showed that 1838–1839 belonged to the same genomic subspecies but that 615–1703 was identified as different but closely related subspecies.

To summarize, WGS of 21 *S. aureus* isolates from different climatic regions in Russia showed the high virulence potential of *S. aureus* in all regions. We observed two groups of *S. aureus* isolates associated with subclinical mastitis, namely, the enterotoxin-positive and enterotoxin-negative groups. The most prevalent factor associated with bovine mastitis in Russia was cytotoxins, including hemolysins and leukocidins. Furthermore, we observed a potential connection of bovine mastitis in Russia with *eta*-positive *S. aureus*. We did not identify MRSA, but the investigated strains may be strongly associated with bovine mastitis due to their multiple combinations of virulence genes, including enterotoxin genes, cytotoxin genes, and bacterial adhesion mediator genes. The limited diversity of enterotoxin genes, which trigger inflammation in the host immune system, in combination with MDR may indicate a long-term chronic form of mastitis (56, 57). Previous antibiotic treatment may transform infection into a chronic subclinical form. MDR may be associated with biofilm formation, which in turn promotes bacterial cell survival during antibiotic treatment and host immune defense mechanisms (58, 59). In previous studies, authors have discussed the problem of long-term, subclinical, and persistent mastitis and its dependence on different virulence factors (60, 61). Grunert et al. (55) showed that high cellular invasiveness of *S. aureus* is associated with chronic infection. Furthermore, high biofilm formation and low level of cellular toxicity may be a supportive mechanism of *S. aureus* to successfully persist and spread in bovines (55).

Although the diversity of virulence factors was investigated in the present study, we did not show expression levels of these factors, which was a limitation of the present study. The expression levels may provide more information about mastitis in Russia. Therefore, our future work will investigate this next step. Our previous study showed that the expression level of some enterotoxins associated with subclinical mastitis in Russia was pure (35).

Studies using genome analysis have facilitated a comprehensive understanding of the epidemiology and genomic repertoire of *S. aureus* from the bovine population and associated this form of mastitis with *S. aureus* as one of the major mastitis pathogens in Russia.

## Data Availability Statement

The datasets generated for this study can be found in the GenBank: WIPL00000000, WIPM00000000, WIPN00000000, WIPO00000000, WIPP00000000, WIPQ00000000, WIPR00000000, WIPS00000000, WIPT00000000, WIPU00000000, WIPV00000000, WIPW00000000, WIPX00000000, WIPY00000000, WIPZ00000000, WIQA00000000, WIQB00000000, WIQC00000000, WIQD00000000, WOUL00000000, and WNKR00000000.

## Ethics Statement

All animal manipulation were provided in accordance with the Instruction for Diagnostics, Therapy and Prevention of mastitis # 13-5-2/1948 (http://gov.cap.ru/home/65/aris/bd/vetzac/document/371.html) and approved by the Department of Veterinary, Ministry of Agriculture of the Russian Federation; and with the Guidelines for the Microbiological Study of Milk and the Secretion of the Udder of Cows for the Diagnosis of Mastitis (https://standartgost.ru/g/pkey-14293740821), Ministry of Agriculture of the Russian Federation.

## Author Contributions

KF and FB designed the study. AS and TD generated the genome assembly and annotation. SS analyzed the phylogeny of isolates. KF, OA, DN, IS, and MS performed the laboratory experiments. KF analyzed the genetic and bioinformatic materials and wrote the paper. All authors read and approved the final manuscript. FB and NZ are supervisors of the project.

### Conflict of Interest

The authors declare that the research was conducted in the absence of any commercial or financial relationships that could be construed as a potential conflict of interest.
