# Supplementary material for: Virulence Factors and Phylogeny of Staphylococcus aureus Associated With Bovine Mastitis in Russia Based on Genome Sequences
Source: Front Vet Sci. 2020 Mar 25;7:135. doi: 10.3389/fvets.2020.00135 (PMC7111254; doi:10.3389/fvets.2020.00135)
Supplement: Supplementary file 1 [file Table_1.doc]

Supplementary Material

Supplementary Table 1. Molecular characteristics of virulence potential and MLST of *S. aureus* isolates associated with subclinical bovine mastitis in Russia.

| **N** | ***S. aureus* isolate** | **Region** | **Entero-toxin gene** | **Entero-toxin like protein gene** | **Super-antigen like protein gene** | **Hemolysin gene** | **Leukocydin gene** | **Adherence genes** | **Exfoliatins gene** | **Immune evasion gene** | **MLST** |
| --- | --- | --- | --- | --- | --- | --- | --- | --- | --- | --- | --- |
| 1 | 70 | Tula | *seg, sei* | *selm, seln, selo, selv* | *set9, 19, 31, 32, 34, 36, 37, 38, 39* | *hla, hlb, hld, hlg* | *lukE/D* | *icaA, icaB, icaC,. icaD, fnbA, fnbB* | *eta* | *spa* | *ST20* |
| 2 | 74 | Tula | *seg, sei* | *selm, seln, selo, selv* | *set9, 19, 31, 32, 34, 36, 37, 38, 39* | *hla, hlb, hld, hlg* | *lukE/D* | *fnbA, fnbB* | *eta* | *spa* | *ST20* |
| 3 | 88 | Tula | *seg, sei* | *selm, seln, selo, selv* | *set9, 19, 31, 32, 34, 36, 37, 38, 39* | *hla, hlb, hld, hlg* | *lukE/D* | *icaA, icaB, icaC,. icaD, fnbA, fnbB* | *eta* | *spa* | *ST20* |
| 4 | 8656 | Moscow | *seg, sei* | *selm, seln, selo, selu, selv* | *set9, 19, 31, 32, 34, 38, 39* | *hla, hlb, hld, hlg* | *lukE/D, lukM/F* | *icaA, icaB, icaC,. icaD, fnbA, fnbB* | *eta* | *spa* | *ST479* |
| 5 | 615 | Perm | *seg, sei* | *selm, seln, selo, selv* | *set9, 19, 31, 32, 34, 36, 37, 38, 39* | *hla, hlb, hld, hlg* | *lukE/D* | *icaA, icaB, icaC,. icaD, fnbA, fnbB* | *eta* | *spa* | *ST20* |
| 6 | 1703 | Moscow | *seg, sei* | *selm, seln, selo, selv* | *set9, 19, 31, 32, 34, 36, 37, 38, 39* | *hla, hlb, hld, hlg* | *lukE* | *icaA, icaB, icaC,. icaD, fnbA, fnbB* | *eta* | *spa* | *ST20* |
| 7 | 1838 | Udmurtia | *seg, sei* | *selm, seln, selo, selv* | *set16* | *hla, hlb, hld, hlg* | *NI* | *icaA, icaB, icaC,. icaD, fnbA, fnbB* | *eta* | *spa* | *ST737* |
| 8 | 1839 | Udmurtia | *seg, sei* | *selm, seln, selo, selv* | *set16, 34, 39* | *hla, hlb, hld, hlg* | *NI* | *icaA, icaB, icaC,. icaD, fnbA, fnbB* | *eta* | *spa* | *ST737* |
| 9 | 23 | Moscow | *NI* | *NI* | *set9, 16, 19, 31, 32, 34, 36, 37, 38, 39* | *hla, hlb, hld, hlg* | *NI* | *icaA, icaB, icaC,. icaD,* | *eta* | *spa* | *ST97* |
| 10 | 8 | Moscow | *NI* | *NI* | *set9, 16, 19, 31, 32, 34, 36, 37, 38, 39* | *hla, hlb, hld, hlg* | *NI* | *icaA, icaB, icaC,. icaD,* | *eta* | *spa* | *ST97* |
| 11 | 812 | Novosibirsk | *NI* | *NI* | *set9, 19, 31, 32, 34, 36, 37, 38, 39* | *hla, hlb, hld, hlg* | *lukE/D* | *icaA, icaB, icaC,. icaD, fnbA, fnbB* | *eta* | *spa* | *ST15* |
| 12 | 724 | Novosibirsk | *NI* | *NI* | *set9, 19, 31, 32, 34, 36, 37, 38, 39* | *hla, hlb, hld, hlg* | *lukE/D* | *icaA, icaB, icaC,. icaD, fnbA, fnbB* | *eta* | *spa* | *ST15* |
| 13 | 1817 | Kirov | *NI* | *NI* | *set9, 16, 19, 31, 32, 34, 36, 37, 38, 39* | *hla, hlb, hld, hlg* | *lukE/D* | *icaA, icaB, icaC,. icaD,* | *eta* | *spa* | *ST97* |
| 14 | 1816 | Kirov | *NI* | *NI* | *set9, 16, 19, 31, 32, 34, 36, 37, 38, 39* | *hla, hlb, hld, hlg* | *lukE/D* | *icaA, icaB, icaC,. icaD,* | *eta* | *spa* | *ST97* |
| 15 | 1813 | Kirov | *NI* | *NI* | *set9, 16, 19, 31, 32, 34, 36, 37, 38, 39* | *hla, hlb, hld, hlg* | *lukE/D* | *icaA, icaB, icaC,. icaD,* | *eta* | *spa* | *ST97* |
| 16 | 1829M | Kirov | *NI* | *NI* | *set9, 16, 19, 31, 32, 34, 36, 37, 38, 39* | *hla, hlb, hld, hlg* | *lukE/D* | *icaA, icaB, icaC,. icaD,* | *eta* | *spa* | *ST97* |
| 17 | 1819M | Kirov | *NI* | *NI* | *set9, 16, 19, 31, 32, 34, 36, 37, 38, 39* | *hla, hlb, hld, hlg* | *lukE/D* | *icaA, icaB, icaC,. icaD,* | *eta* | *spa* | *ST97* |
| 18 | 1835M | Kirov | *NI* | *NI* | *set9, 16, 19, 31, 32, 34, 36, 37, 38, 39* | *hla, hlb, hld, hlg* | *lukE/D* | *icaA, icaB, icaC,. icaD,* | *eta* | *spa* | *ST97* |
| 19 | 18131 | Kirov | *NI* | *NI* | *set9, 16, 19, 31, 32, 34, 36, 37, 38, 39* | *hla, hlb, hld, hlg* | *lukE* | *icaA, icaB, icaC,. icaD,* | *eta* | *spa* | *ST97* |
| 20 | 187M | Udmurtia | *NI* | *NI* | *set9, 16, 19, 31, 32, 34, 36, 37, 38, 39* | *hla, hlb, hld, hlg* | *lukE/D* | *icaA, icaB, icaC,. icaD,* | *eta* | *spa* | *ST97* |
| 21 | 1709 | Saratov | *NI* | *NI* | *set9, 16, 19, 31, 32, 34, 36, 37, 38, 39* | *hla, hlb, hld, hlg* | *lukE* | *icaA, icaB, icaC,. icaD* | *eta* | *spa* | *ST97* |

NI – non identified
